# Using hospital discharge data for determining neonatal morbidity and mortality: a validation study

**DOI:** 10.1186/1472-6963-7-188

**Published:** 2007-11-20

**Authors:** Jane B Ford, Christine L Roberts, Charles S Algert, Jennifer R Bowen, Barbara Bajuk, David J Henderson-Smart, the NICUS group

**Affiliations:** 1 Perinatal Research Group, Kolling Institute of Medical Research, University of Sydney, New South Wales 2065, Australia.; 2 Royal North Shore Hospital, St Leonards, New South Wales 2065, Australia.; 3 Centre for Perinatal Health Services Research, University of Sydney, New South Wales 2006, Australia.

## Abstract

**Background:**

Despite widespread use of neonatal hospital discharge data, there are few published reports on the accuracy of population health data with neonatal diagnostic or procedure codes. The aim of this study was to assess the accuracy of using routinely collected hospital discharge data in identifying neonatal morbidity during the birth admission compared with data from a statewide audit of selected neonatal intensive care (NICU) admissions.

**Methods:**

Validation study of population-based linked hospital discharge/birth data against neonatal intensive care audit data from New South Wales, Australia for 2,432 babies admitted to NICUs, 1994–1996. Sensitivity, specificity and positive predictive values (PPV) with exact binomial confidence intervals were calculated for 12 diagnoses and 6 procedures.

**Results:**

Sensitivities ranged from 37.0% for drainage of an air leak to 97.7% for very low birthweight, specificities all exceeded 85% and PPVs ranged from 70.9% to 100%. In-hospital mortality, low birthweight (≤1500 g), retinopathy of prematurity, respiratory distress syndrome, meconium aspiration, pneumonia, pulmonary hypertension, selected major anomalies, any mechanical ventilation (including CPAP), major surgery and surgery for patent ductus arteriosus or necrotizing enterocolitis were accurately identified with PPVs over 92%. Transient tachypnea of the newborn and drainage of an air leak had the lowest PPVs, 70.9% and 83.6% respectively.

**Conclusion:**

Although under-ascertained, routinely collected hospital discharge data had high PPVs for most validated items and would be suitable for risk factor analyses of neonatal morbidity. Procedures tended to be more accurately recorded than diagnoses.

## Background

Population health data provide a powerful tool for investigating health outcomes and assessing health interventions[1,2]. Hospital discharge data represent a potential source of population-based data on neonatal health outcomes and associated maternal characteristics and conditions. Despite widespread use of neonatal hospital discharge data [3-5], there are few published reports on the accuracy of population health data with neonatal diagnostic or therapeutic intervention codes[6]. Validation of neonatal outcomes in Australian hospital discharge data has been limited to diagnosis-related codes[7], although health interventions and procedures are generally better reported in population health data and may be better markers of morbidity [8-10]. Furthermore previous validation studies of neonatal outcomes have been random samples from the entire birth population, and have therefore included few high risk babies so the assessments of severe morbidity reporting has lacked precision[7,11].

Regionalized maternity care, in which high risk mothers and/or infants are transferred to higher levels of care, aims to ensure all women and their babies get the care they require[12]. In Australia, the highest level of perinatal care is provided by perinatal centres which provide both tertiary obstetric care and neonatal intensive care. While there is currently a statewide New South Wales (NSW) audit of babies who are admitted to neonatal intensive care units (NICU) for selected reasons [13], there is no population-based reporting on all babies admitted to NICU or who suffer a major morbidity.

Accurate population measures of neonatal morbidity would enable assessment of the quality of all levels of care[14]. Establishing the accuracy of morbidity reporting among high risk babies in population data (compared with available audit data) would allow us to maximise the usefulness of population health data in tracking changes in the high-risk neonatal population and facilitate further study on the effectiveness of regionalized maternity care using linked population data. Of particular interest is whether the babies identified as having a neonatal morbidity or procedure in the population data genuinely have a serious morbidity associated with their birth. This measure, the positive predictive value (PPV), is used to quantify the usefulness of routinely collected population health data[15]. Therefore the aim of this study was to assess the accuracy of the routinely collected hospital discharge data in identifying neonatal morbidity among high risk infants during the birth admission to hospital compared with a statewide audit (gold standard) of selected NICU admissions.

## Methods

NSW is the most populous state in Australia with ~6.3 million people and 86,000 births per annum, of which <0.5% are planned home births[13,16]. The state has an area of 800,000 km^2 ^and population densities range from 2,000 persons/km^2 ^in the coastal cities to <0.1 person/km^2 ^in the most remote areas[16].

Data for this study were obtained from three different datasets. The NSW Midwives Data Collection (MDC) is a legislated population-based surveillance system covering all births in NSW public and private hospitals ≥ 20 weeks gestation or ≥ 400 g birthweight, as well as homebirths and includes data on maternal characteristics, pregnancy, labour, delivery and infant outcomes [13]. The NSW Admitted Patient Data Collection (APDC) is a census of all NSW inpatient hospital discharges, coded according to the 9th revision of the International Classification of Diseases, Ninth Revision, Clinical Modification (ICD9) with up to 11 diagnoses and 10 procedures coded per hospital stay during the study period[17]. Hospital coders use all available information in the medical record to code diagnoses and procedures. The Neonatal Intensive Care Units (NICUS) Data Collection is an audit of selected neonatal admissions to a NSW Neonatal Intensive Care Unit for one of the following NICUS registration criteria: gestational age less than 32 weeks, birthweight ≤ 1500 gms, mechanical ventilation for 4 hours or more, continuous positive airways pressure (CPAP) for 4 hours or more, and/or major surgery (opening of a body cavity)[13]. NICUS includes neonatal diagnoses and procedures which were abstracted by 8 clinical nurse specialists with neonatal intensive care experience using standard data abstraction forms and definitions in each of 8 neonatal intensive care units during the study period[13]. Data are retrospectively abstracted from medical records and cover details of the first NICU admission, the hospital babies were transferred from and to, selected diagnoses and procedures that were performed during the first or subsequent NICU admissions, before discharge home. For example, if a baby is transferred from a perinatal centre NICU to a children's hospital for surgery, the surgical procedures are recorded in a single NICUS record. NICUS data were considered to be the 'gold standard' in this validation since it is abstracted by trained professionals located in each of the neonatal intensive care units.

### Record linkage

For the years 1994–1996, NICUS records were linked to the APDC via MDC birth records. Direct linkage of NICUS and APDC records has not been undertaken, however NICUS and MDC records have previously been linked as have MDC-APDC records. The NSW Department of Health uses probabilistic record linkage which has been described elsewhere [18]. Only de-identified data were available to researchers.

When the datasets were limited to babies born in perinatal centres in NSW there were 68 956 birth records eligible for linkage (of which 2927 potentially met the NICUS registration criteria) and 2690 NICUS records. Given that some babies will die before NICUS admission and others will be ventilated for less than four hours it is not surprising that there were 237 additional birth records identified. After linkage there were 108 (3.7%) records that partially linked (NICUS linked to a MDC record but the MDC did not link to an APDC record), and 150 (5.1%) NICUS records that did not link to an MDC record, leaving 2432 linked records available for analysis.

### Diagnoses and procedures available for assessment of accuracy and reliability

Diagnoses available for comparison on both NICUS and the APDC included: very low birthweight, death in hospital, intraventricular hemorrhage (IVH), retinopathy of prematurity (ROP), necrotizing enterocolitis (NEC) and selected major anomalies including anomalies of the diaphragm, abdominal wall anomalies, spina bifida, tracheo-oesophageal fistula (TOF) stenosis or atresia, and tetralogy of fallot. We also assessed any brain hemorrhage, respiratory distress syndrome (RDS), transient tachypnea of the newborn (TTN), meconium aspiration, pneumonia and pulmonary hypertension (Table 1) although these variables are not collected in the same way on each data set (Table 2). NICUS data for some of these variables involved reporting of a primary diagnosis, and consequently not all diagnoses were necessarily recorded in these data. Procedures available for assessment included patent ductus arteriosus (PDA) surgery, NEC surgery, major surgery (using the NICUS definition of opening of a body cavity), drainage of an air leak, continuous positive airways pressure (CPAP) and any mechanical ventilation (including CPAP). If no diagnosis or procedure was recorded it was presumed to be absent. Gestational age in weeks was available on the MDC but not routinely reported on APDC data. See Appendix 1 for a comparison of the collection and format of the dichotomous variables compared on both datasets.

**Table 1:** Accuracy of reporting in the NSW Inpatient Statistics Collection, New South Wales, Australia, 1994–1996

**Description**	**NICUS data N**	**APDC data** **N**	Both data sets* N	**Sn %** **(95% CI)**	**Sp %** **(95% CI)**	**NPV %** **(95% CI)**	**PPV %** **(95% CI)**
**Diagnoses**							
Death in birth admission	289	261	260	90.0 (85.9–93.2)	100 (99.7–100)	98.7 (98.1–99.1)	99.6 (97.9–100)
Very low birthweight 400–1499 g	1,242	1,216	1213	97.7 (96.7–98.4)	99.7 (99.2–99.9)	97.6 (96.5–98.4)	99.8 (99.3–99.9)
Intra-ventricular hemorrhage	394	238	205	52.0 (47.0–57.1)	98.4 (97.7–98.9)	91.4 (90.1–92.5)	86.1 (81.1–90.3)
Any brain hemorrhage‡	400	281	251	62.8 (57.8–67.5)	98.4 (97.7–98.9)	92.4 (91.1–93.5)	89.3 (85.1–92.7)
Retinopathy of prematurity	273	169	157	57.5 (51.4–63.4)	99.4 (99.0–99.7)	94.9 (93.9–95.7)	92.9 (87.9–96.3)
Respiratory distress syndrome‡	1205	1022	991	82.2 (80.0–84.4)	92.4 (89.4–94.8)	63.9 (59.8–67.7)	97.0 (95.7–97.9)
Transient tachypnea‡	347	206	146	42.1 (36.8–47.5)	85.3 (81.5–88.6)	63.5 (59.3–67.5)	70.9 (64.2–77.0)
Meconium aspiration‡	56	40	38	67.9 (54.0–79.7)	99.5 (98.2–99.9)	95.8 (93.4–97.5)	95.0 (83.1–99.4)
Pneumonia‡	29	15	14	48.3 (29.4–67.5)	99.8 (98.6–100)	96.5 (94.2–98.0)	93.3 (68.1–99.8)
Pulmonary hypertension‡	53	34	34	64.2 (49.8–79.9)	100 (99.1–100)	95.6 (93.2–97.3)	100 (89.7–100)
Necrotizing enterocolitis	76	55	47	61.8 (50.0–72.8)	99.7 (99.3–99.9)	98.8 (98.3–99.2)	85.5 (73.3–93.5)
Selected major anomalies†	41	42	39	95.1 (83.5–99.4)	99.9 (99.6–100)	99.9 (99.7–100)	92.9 (80.5–98.5)
**Procedures**							
Any mechanical ventilation (including CPAP)	2,023	1,552	1540	76.1 (74.2–78.0)	97.1 (94.9–98.5)	45.1 (41.8–48.5)	99.2 (98.7–99.6)
Cont. positive airways pressure	1055	785	691	65.5 (62.5–68.4)	93.2 (91.7–94.4)	77.9 (75.8–79.9)	88.0 (85.5–90.2)
Drainage of air leak	135	60	50	37.0 (28.9–45.8)	99.6 (99.2–99.8)	96.4 (95.6–97.1)	83.3 (71.5–91.7)
Patent ductus arteriosus surgery	19	18	18	94.7 (74.0–99.9)	100 (99.8–100)	100 (99.8–100)	100 (81.5–100)
Necrotizing enterocolitis surgery	17	14	14	82.4 (56.6–96.2)	100 (99.8–100)	99.9 (99.6–100)	100 (76.8–100)
Major surgery	77	74	70	90.9 (82.2–96.3)	99.8 (99.6–100)	99.7 (99.4–99.9)	94.6 (86.7–98.5)

Sn = Sensitivity, Sp = Specificity;* N = 2,432;

†Selected major anomalies included diaphragm anomalies, abdominal wall anomalies, spina bifida, tracheo-esophageal fistula and tetralogy of fallot. ‡ These diagnoses may be under-recorded in the NICUS data since only a primary diagnosis could be recorded (see Table 2)

**Table 2:** Comparison of NICUS variable codes and ICD9 codes available in the Admitted Patient Data Collection, New South Wales, Australia, 1994–1996

	**NICUS variable codes**	**Available ICD9 codes**
**Diagnoses**		
Intraventricular hemorrhage (IVH) and other brain hemorrhages	IVH was coded as YES (grades 1–4) or NO (none/not examined) A *single *head ultrasound (HUS) abnormality can be recorded and coded: 0:None; 1:Cerebral oedema; 2:Subarachnoid hemorrhage; 3:Periventricular echogenicity; 4:Cerebellar hemorrhage; 5:Subdural hemorrhage; 6:Extradural hemorrhage; 7:Other; 8 Not examined.	772.1 Neonatal IVH 772.2 Neonatal subarachnoid hemorrhage 767.0 Subdural and cerebral hemorrhages 772.1, 772.2 or 767.0 any brain hemorrhage
	Any brain hemorrhage was coded YES where IVH = YES or HUS in (2,4,5,6) and NO where HUS abnormality of 'none' or 'not examined'.	
Respiratory diagnoses	A *single *'primary respiratory diagnosis' can be recorded and coded: 0:None; 1:Transient tachypnea of the newborn (TTN); 2:Respiratory distress syndrome (RDS); 3:Meconium aspiration; 4:Immature lung; 5:Apnea; 6:Congenital anomaly; 7: Pulmonary hypertension; 8:Pneumonia; 9:Perinatal surgery; 10:Other; or 11:Unknown	769 Respiratory distress syndrome (neonatal) 770.6 Transient tachypnea of the newborn 770.1 Meconium aspiration syndrome 770.0 Congenital pneumonia 480–486 Pneumonia 416.0 Pulmonary hypertension
	A *single *'main indication for initial ventilation" can also be recorded and coded 1–7 as above or; 8:Infection (any site); 9:Asphyxia; 10: Cardiac disorder; 11:Post-surgery; 12:Other	
	Any diagnosis of transient tachypnea, respiratory distress syndrome, meconium aspiration, pulmonary hypertension or pneumonia was coded YES and 'primary respiratory diagnosis of 'none' coded NO	
Retinopathy of prematurity (ROP)	ROP was coded as YES (grades 1–4 or treatment for ROP) or NO (none/not examined)	362.21 Retrolental fibroplasia 362.10 Retinopathy unspecified 14.2 Destruction of chorioretinal lesion 36.21, 362.10 or 14.2 coded as ROP
Necrotizing enterocolitis (NEC)	NEC coded as YES (clinically diagnosed or proven radiologically/at surgery) or NO (None)	777.5 Necrotizing enterocolitis in newborn
Selected major anomalies	Recorded using ICD9 diagnosis codes for any of the following: anomalies of diaphragm, anomalies of abdominal wall, spina bifida, tracheo-oesophageal fistula (TOF), oesophageal atresia and stenosis and tetralogy of fallot (see next column for ICD codes)	756.6 Diaphragm anomalies 756.7 Abdominal wall anomalies 741 Spina bifida 750.3 TOF, oesophageal atresia and stenosis 745.2 Tetralogy of fallot
**Procedures**		
Continuous positive airway pressure (CPAP)	Recorded using 4 variables indicating number of days of endotracheal tube CPAP, nasopharyngeal CPAP, continuous nasal CPAP and intermittent nasal CPAP: 1 or more days of any of these recorded as YES, 0 days recorded as NO	93.90 Continuous positive airway pressure (CPAP)
Mechanical ventilation (including CPAP)	Mechanical ventilation or CPAP, YES or otherwise NO	93.90 Continuous positive airway pressure (CPAP) 93.91 Intermittent positive airway pressure breathing 96.7 Continuous mechanical ventilation 93.90, 93.91, or 96.7 for any mechanical ventilation
Drainage of air leak	Recorded as air leak requiring drainage YES or NO	34.04 Insertion of intercostal catheter for drainage
Surgery for patent ductus arteriosus (PDA)	PDA surgery coded as YES or NO	38.85 Ligation of PDA
Surgery for necrotizing enterocolitis (NEC)	NEC surgery coded as YES or NO	777.5 Necrotizing enterocolitis in newborn 45.6–46.5 Operations on the small or large intestine 43.19 Other gastrostomy 54.11, 54.19 Laparotomy 54.59 Lysis of peritoneal adhesions 77.5 *and *45.6–46.5, 43.19, 54.11, 54.19, or 54.59 for NEC surgery

### Analysis

Linked MDC-APDC-NICUS records of babies born in perinatal centres were compared with non-linking birth records that fulfilled the NICUS registration criteria using the chi-square test and a significance level of 0.05. MDC gestational age was used for assessing of NICUS criteria. Among the linked records the reporting of diagnoses and procedures on the APDC was assessed by determining the sensitivity, specificity, positive predictive values (PPV) and negative predictive values (NPV) with exact binomial confidence intervals[19], using the NICUS as the 'gold standard'. *Sensitivity *was the percentage of babies with a diagnosis/procedure identified in NICUS for whom the same diagnosis/procedure was reported on the hospital discharge data, therefore denoting the completeness of identification by hospital discharge data. *Specificity *was the percentage of babies without a diagnosis/procedure on NICUS who were correctly reported as not having it in the hospital discharge data. *Positive predictive value *was the percentage of babies' reports in the hospital discharge data with the same diagnosis/procedure reported in the NICUS audit. *Negative predictive value *was the percentage of babies reported as not having a diagnosis/procedure in hospital discharge data for whom a NICUS report was also absent. The PPV and NPV are measures of the accuracy of the hospital data. *Positive likelihood ratios *were also reported as a measure of accuracy which takes into account the prevalence of a condition/procedure. Likelihood ratios of above 10 are considered to provide strong evidence to rule in diagnoses in most circumstances [20], with the larger likelihood ratios indicating greater likelihood of correct reporting if the diagnosis/procedure is present in the hospital discharge data. In using population-based data for risk factor analyses it is important that identified cases are true cases (high PPV). Analyses were carried out using SAS v9.1. The study protocol was approved by the Sydney South West Area Health Service Ethics Review Committee.

## Results

During 1994–1996, there were 2927 babies born in perinatal centres in NSW with linked MDC-APDC birth records that potentially met the NICUS registration criteria and 2432 (83%) that actually linked to a NICUS record. Compared with records that linked to NICUS, those that did not link were significantly (p < 0.0001) more likely to include babies who: died on the day of birth (11% vs 2%) were born at ≥ 32 weeks gestation (26% vs 11%), birthweight ≥ 1500 g (38% vs 25%) or transferred to another perinatal centre (36% vs 0.2%).

Of the 2432 babies who had birth records that linked to a NICUS record, 1591 (65.4%) were registered in NICUS because they were ≤ 1500 g or <32 weeks, 831 (34.2%) for any mechanical ventilation (including CPAP) and 10 (0.4%) for major surgery. Table 1 summarises the sensitivity, specificity, and PPV for the morbidities and procedures that could be compared in NICUS and the APDC.

There was a wide range of sensitivities for the reporting of neonatal morbidities, ranging from 42% for TTN to 98% for very low birthweight (Table 1). Of the 12 morbidities compared, all but one (TTN) had PPVs over 80% and eight were over 90% including death in hospital, very low birthweight, RDS, meconium aspiration, pneumonia, pulmonary hypertension, ROP and the selected major anomalies (Table 1).

The selected congenital anomalies were well identified. There was perfect agreement between the datasets for TOF (n = 5) and spina bifida (n = 5), with high PPVs for other anomalies ranging from 83% for tetralogy of fallot (n = 5) to 87% for abdominal wall anomalies (n = 14) and 94% for anomalies of the diaphragm (n = 16).

Four out of the six procedures validated had PPVs of 95% or above. Although ascertainment of any mechanical ventilation (including CPAP) was only 76%, the PPV was extremely high at 99% indicating false positives rarely occur (Table 1). In contrast, CPAP as a specific procedure was less accurately recorded with a PPV of 88% and a sensitivity of 66%. However, an additional 165 cases of CPAP were identified in the hospital data as having mechanical ventilation of some kind, so that its ascertainment rose to 81% within this broader category. Drainage of an air leak had a low ascertainment rate. The identification of major and specific surgeries was very accurate with PPVs of 95% or more.

Positive likelihood ratios were above 10 for all diagnoses except transient tachypnea and all procedures except CPAP. The range of values above 10 were from 10.8 for respiratory distress syndrome to 951 for selected major anomalies.

## Discussion

In general, the findings of this validation study are consistent with other validation studies that compare routinely collected population health data with medical records [15,21,22]. Diagnoses and procedures tend to be under-ascertained in population health data[8], but the majority of the cases that are identified are true cases with PPVs over 80%. Procedures are generally more accurately reported than diagnoses[9,11,22], with only two procedures with a PPV below 95% in this study.

It is important to note that validation of data from a high risk population presents particular challenges. Preterm and very low birthweight babies are likely to have multiple diagnoses and procedures recorded. While hospital discharge data are structured to record multiple diagnoses and procedures, audit data in some cases involves assessments of primary diagnoses, (for example, primary respiratory diagnosis) and consequently *not all *diagnoses are necessarily recorded for 6 of the 18 items validated (see Table 1). In addition, the widespread use of mechanical ventilation and monitoring in the NICU population may result in brief interventions (<4 hours) not being recorded in audit data (in an attempt to only include true respiratory pathology) whereas administrative requirements dictate the need for recording of all interventions in the hospital discharge data.

Previous research has shown that demographic variables such as infant sex, birthweight, and in Australia gestational age, are well recorded[11,23]. This is the first time birthweight (as identified in the hospital discharge data) has been validated, with encouraging results. Although restricted to validation of very low birthweight, a PPV of over 99% indicates that this variable can be used to identify high risk babies in the hospital discharge data. As the NICUS registration criteria (birthweight ≤ 1500 g, mechanical ventilation and major surgery) were identified with a high probability (PPVs > 94%) in the APDC and gestational age is well reported in the MDC[11], these babies can be readily identified from the linked birth data.

Some diagnoses, such as TTN and brain hemorrhages, had lower PPVs. This is likely to be related to reporting differences between the datasets. NICUS may under-enumerate some conditions when it allows only a single diagnosis, such as primary respiratory diagnosis or head ultrasound abnormality; additional related diagnoses are not recorded. For example a baby may have both meconium aspiration and pulmonary hypertension or a subarachnoid hemorrhage and cerebral oedema, but only one of these could be coded. In contrast the APDC could capture all diagnoses and therefore may include true cases that NICUS does not. Furthermore, TTN may be difficult to diagnose and may resolve with minimal intervention, and as such, may be less likely to be reported. Medical record coding is better where a major intervention or procedure occurs [9].

Other diagnoses could not be compared in the two datasets because there were no specific ICD9 codes (eg. immature lung, periventricular echogenicity and cerebellar hemorrhage) or there were only very general, non-specific codes that included other diagnoses. For example apnea is included in the ICD9 code '770.8 Other respiratory problems at birth' which also includes cyanotic attacks, respiratory distress and respiratory failure not otherwise specified.

Despite differences between the datasets in the recording of any mechanical ventilation, the PPV was 99%. One of the NICUS registration criteria is mechanical ventilation for ≥ 4 hours but it is not possible to impose this duration limit on APDC data. The APDC records any non-operating theatre mechanical ventilation so it is entirely possible that infants reported as receiving mechanical ventilation in the APDC, but not in the NICUS data, did receive ventilation but of less than four hours duration. The more specific code of CPAP was less accurately reported than the codes that capture any mechanical ventilation which included CPAP. This is consistent with other studies involving hospital data which have shown that broader categories have greater validity than very specific categories[7,24].

The availability of record linkage only for the birth admission limited our ability to validate the reporting of surgical procedures. Most neonatal surgery is performed at children's hospitals in which there are no births[25]. Therefore there are, and rightly should be, few cases of surgical procedures reported in this study population. Nevertheless, the surgical procedures that could be assessed were identified with an extremely high level of accuracy especially for the highly specific procedure of ligation of a PDA.

It is unclear why air leak requiring drainage had a markedly lower sensitivity (38%) than other procedures, although most of the cases identified in the APDC (83%) were true cases according to NICUS. Major surgical procedures conducted in an operating theatre may be better ascertained in hospital discharge records than procedures that were probably undertaken in the NICU as a component of neonatal intensive care, such as insertion of a chest drain or mechanical ventilation.

Potential criticisms of the study include variations in recording of diagnoses/procedures between the two datasets, possible under-ascertainment of selected NICUS variables and the use of data that are more than ten years old. Linkage of MDC-APDC-NICUS data was only conducted for the three year period covered by this study and is unlikely to be extended to cover more recent years. There have been changes in the risk profile of women giving birth[13](eg higher proportions of pregnancies with a history of cesarean delivery, advancing maternal age) potentially resulting in an increased proportion of complicated births since 1996. At the same time there has been increased survival of preterm infants born at earlier gestational ages [26]. However, these trends have probably affected the numbers and proportions of babies with particular diagnoses (eg. retinopathy of prematurity), but are unlikely to have negatively affected the recording of these diagnoses. If anything such potentially increased incidence would be likely to improve reporting [27].

### Implications for population-based analyses

Hospital discharge data are an important resource for health surveillance, informing health service provision and risk factor analyses. While under-ascertainment affects the reporting of the prevalence of diagnoses and procedures and thus will result in under-reporting of baby outcomes, it may have little impact on the analysis of risk factors because the number of non-reported cases (false negatives) is very small relative to the number of non-cases (true negatives) in population data[28]. For example, if only 62% of NEC diagnoses are identified in a population of 100,000 live births with an expected incidence of 110 babies with NEC [29], the 42 missed cases would not impact a risk factor analysis if they were included in the 99,800 babies who did not have NEC. Consequently moderately low sensitivities for severe morbidity reporting of diagnoses such as NEC and ROP does not preclude their use in risk factor analyses in population data. This assumes, however, that there is non-differential misclassification of cases and non-cases with respect to determinants and confounders, and in terms of severity. These are important issues for consideration given that hospital discharge data appears to capture more severe cases [30,31]and may over-estimate associations with outcomes [30].

Given the high-risk study population in this study and the impact that prevalence has on PPVs, the predictive values for common diagnoses/procedures observed in this study, such as very low birthweight, respiratory distress syndrome and mechanical ventilation, will not necessarily be transferable to other studies. In a low-risk population we would be more sure that no such recorded diagnosis/procedure indicated true absence, and less sure that a positive result really indicated a condition/procedure was present [32]. Nevertheless, likelihood ratios indicate these items will be useful in wider hospital discharge data.

As expected, more records in the linked birth admissions at perinatal centres fulfilled the NICUS registration criteria than linked with a NICUS record. This is because the perinatal centre population data includes labour ward deaths[33], babies transferred to other NICUs and larger or more mature babies who were ventilated for < 4 hours. This is a reminder that NICUS is an audit of selected NICU admissions and cannot provide a population measure of neonatal morbidity and mortality. While differential recording accuracy may occur between low-risk and high-risk populations[34], we would expect recording to be consistent within the high-risk population. Therefore, we would expect these findings would be generalizable to other high risk babies not included in our study (eg. babies transferred to another NICU, term babies with meconium aspiration). Reassuringly, our results are comparable with an audit of a random sample of 500 babies conducted in 1999–2000 which, though based on small numbers of cases, similarly showed some under-reporting but high PPVs for intraventricular hemorrhage (n = 4), respiratory distress syndrome (n = 14), meconium aspiration (n = 4) and congenital anomalies (n = 49) among the general neonatal population[7].

## Conclusion

Assuming that the accuracy of the neonatal morbidity reporting is generalizable to other high risk babies in the hospital discharge data (not included in our study), a composite measure of neonatal morbidity (based on identification from a prescribed list of validated morbidities) could be developed and applied to all births and not just those fulfilling the NICUS registration criteria. However, to capture all neonatal morbidity would require birth admission data to be linked to subsequent admissions including those following transfer to a perinatal centre or children's hospital[35].

## Competing interests

The author(s) declare that they have no competing interests.

## Authors' contributions

JF and CR conceived and designed the study and undertook analysis and interpretation, CA participated in analysis and interpretation, JB and DHS participated in interpretation and BB acquired and linked data and participated in interpretation. All authors read and approved the final manuscript.

## Pre-publication history

The pre-publication history for this paper can be accessed here:


